# A Novel Infrared Temperature Measurement with Dual Mode Modulation of Thermopile Sensor

**DOI:** 10.3390/s19020336

**Published:** 2019-01-15

**Authors:** Chih-Hsiung Shen, Shu-Jung Chen, Yi-Ting Guo

**Affiliations:** Department of Mechatronics Engineering, National Changhua University of Education, Changhua City 50074, Taiwan; sjchen@cc.ncue.edu.tw

**Keywords:** thermopile, switching, modulation, CMOS-MEMS

## Abstract

Superior to the traditional infrared temperature sensing architecture including infrared sensor and thermistor, we propose a novel sensing approach based on a single thermopile sensor with dual modes modulation. A switching and sensing circuit is proposed and realized with a chopper amplifier AD8551 and p-channel MOSFET (PMOS) for switching between detection of thermal radiation and the target and the ambient temperature for compensation. The error of target temperature after temperature compensation is estimated at less than 0.14 °C.

## 1. Introduction

The rise of the semiconductor industry has enabled a large number of sensors to be explored with more intelligent features. With the success of micro-electromechanical and circuits technology, many micro-sensors and micro-actuators have gradually been realized into micro-electromechanical products. Infrared thermal sensors are widely developed and used for home and industrial applications since the variety of features have made great progress, including in the sensitivity of sensor and the reduction of size embedded into the associated facilities and instrument. Moreover, the low power consumption and fast response bring the thermal micro-sensor technology into a new era of multi-sensors and multiple sensing with hybrid-sensor concept [1] beyond the traditional technology.

Besides infrared temperature sensing, the thermal sensors are also excellent candidates intended for measurements of atmospheric pressures and vacuum sensors to explore many important applications [2] based on the integration of complementary metal-oxide-semiconductor (CMOS) with micro-electromechanical systems (MEMS) technology. Especially for the CMOS-MEMS technology, thermopile-based MEMS vacuum sensors [3] give a new approach with mature integration of MEMS sensors and circuits and bring advantages with wide pressure range. Based on the Seebeck effect, the thermoelectric sensors are also studied and developed for gas sensors, 2D inertial sensors, flow sensors, etc. [4,5,6], which is proven to be an excellent sensing element for most of the sensing related to the thermal properties [7,8].

The CMOS-MEMS thermopile sensor has revealed great advantages beyond the traditional thermal sensors. Since the thermopile works as a thermoelectric sensor and the material properties of thermoelectric sensor are sensitive to temperature [9], it can be considered to be both a thermoelectric sensor and thermistor. Furthermore, the novel concept is developed and realized broadly for many applications and achieves tremendous results [10]. For our previous research work, we have shown a single-element thermopile flow sensor acting as a sensor and transducer can be biased to heating and also output a voltage related to the infrared radiation. Kyle Clocker et al. [11] had also shown that by separating heating and measurement into two steps in time, it will bring an advantage with integrated circuits to reveal a method for measuring the speed of flow using a single transducer element with a new control algorithm with two phases.

In this research, the proposed dual mode modulation of sensor is superior to the traditional infrared temperature sensing systems with two kind of sensors. Besides infrared sensing, the thermopile made of polysilicon could perform the function of thermistor because its resistance is a function of temperature. Therefore, it reveals a novel infrared temperature sensing approach based on a single thermopile sensor with dual modes modulation which play the roles of both infrared sensing and ambient temperature sensor. A switching circuit is proposed and realized with a chopper amplifier AD8551 and p-channel MOSFET (PMOS) to switch between the detection of thermal radiation from the target and the ambient temperature for compensation.

## 2. Working Principle and Design for Dual Mode Modulation

**Figure 1:**
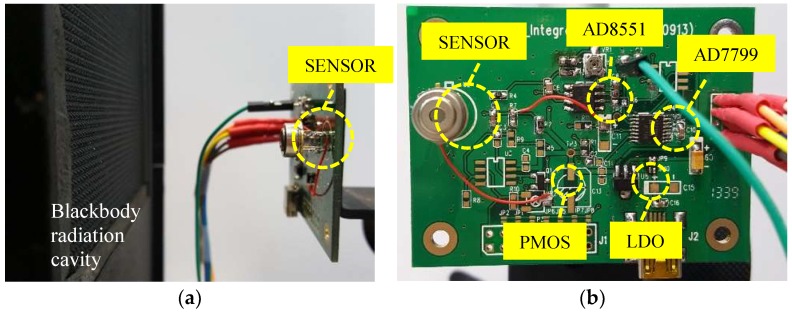
(**a**) Experimental setup; (**b**) Proposed switching circuit board of dual mode.

In general, the thermal radiation temperature measurement uses an infrared thermal radiation sensor to measure the amount of thermal radiation that is incident on the sensor. It also includes a thermistor to measure the ambient temperature and calculate the amount of external radiation. The difference of the two radiations is net heat radiation, and the target temperature can be estimated. This study used a black body radiation cavity to generate a target temperature and placed the sensor 4 cm in front of the cavity, as shown in Figure 1a.

The sensor of proposed thermopile with TO-5 package is connected to a dual mode switching circuit. In this circuit, the power is supplied and regulated by a source circuit containing a low-dropout regulator (LDO), and the switching circuit is implemented by a PMOS.The output signal of thermopile sensor is delivered to an amplifier AD8551 with low offset and an Analog-to-Digital Converter (ADC) 24-bit, 3-channel AD7799 which transfers it into the digital signal, as shown in Figure 1b.

**Figure 2:**
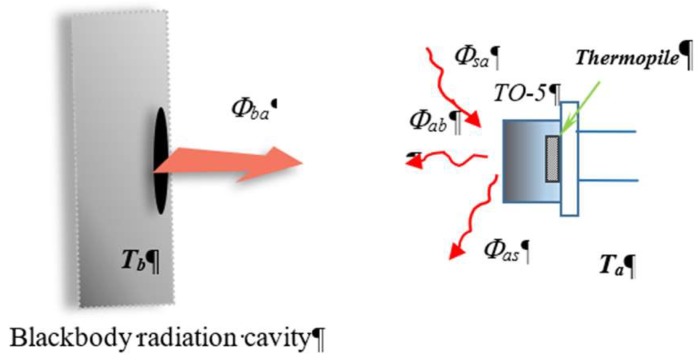
Configuration diagram of infrared measurement with dual modes.

The mathematical model of thermal radiation measurements is expressed as Equations (1) to (3) below, which reveal the relationship of infrared radiation to ambient temperature for infrared temperature sensing. In general, it is necessary to consider the ambient temperature compensation for target temperature measurement which means a thermistor sensor for ambient temperature sensing is required. Since the output voltage of thermopile sensor corresponds to the net infrared radiation energy from the radiation exchange between the blackbody, the thermopile sensor, and the surrounding environment, the algorithm and sensors of measurement are complicated, as shown in Figure 2. Usually, it is simplified to assume a thermal equilibrium for the temperature of thermopile and environment with $\Phi_{as}\approx \Phi_{sa}$. $\Phi_{as}$ stands for the radiation from the sensor to the surrounding environment, and the radiation $\Phi_{sa}$, vice versa. In Equation (1), the net infrared radiation energy received for thermopile is expressed as ΔΦ, where it finally includes the radiation $\Phi_{ba}$ from the target to sensor, and the radiation $\Phi_{ab}$, vice versa. It is also well known that the radiation can be expressed as the exchange of radiation from Equations (2) to (5) based on the Stefan–Boltzmann law,

(1) $$\Delta \Phi =\Phi_{ba}+\Phi_{sa}-\Phi_{ab}-\Phi_{as},$$

(2) $$\Phi_{ba}={Α}_{ba}\varepsilon_{b}\sigma {Τ}_{b}{}^{4},$$

(3) $$\Phi_{sa}={Α}_{sa}\varepsilon_{a}\sigma {Τ}_{a}{}^{4},$$

(4) $$\Phi_{ab}={Α}_{ab}\varepsilon_{a}\sigma {Τ}_{a}{}^{4},$$

(5) $$\Phi_{as}={Α}_{as}\varepsilon_{a}\sigma {Τ}_{a}{}^{4},$$

where the geometric factor is represented as Α, which describes the optical factors of various elements presented in the optical path of infrared radiation from the target to sensor. The optical factors of element include the field of view of the sensor for the target, the optical characteristics of filter, size of sensor, and so on. The field of view is described as the solid angle from the source to the sensor, the optical characteristics of filter means the transmitted spectrum of infrared radiation and size of sensor will decide the received infrared power. The emissivity of the surface of a material is its effectiveness in emitting and receiving energy as thermal radiation, especially for the target and sensor. The emissivity of target and sensor material ε are also the dominated factors for the sensitivity of sensing system.

## 3. Design and Fabrication of Thermopile Sensor

**Figure 3:**
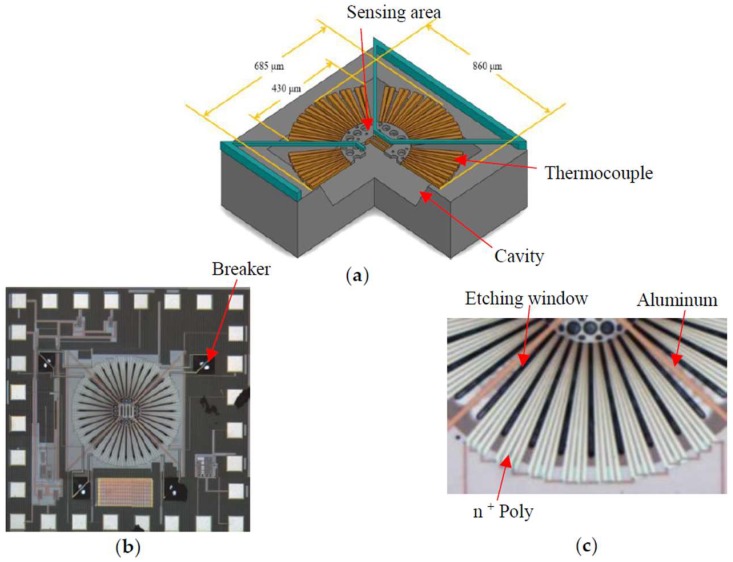
(**a**) Schematic drawing of thermopile structure; (**b**) Full chip after fabrication; (**c**) A quarter of the thermopile.

For the investigation of dual mode modulation of infrared sensor, a thermopile is fabricated by using the standard TSMC 0.35 μm 2P4M (2-polysilicon 4-metal) CMOS process and the subsequent MEMS post-processes. Figure 3 shows the design of thermopile elements, the whole chip after fabrication, and a quarter of the thermopile. The thermopile element structure is centrally symmetrical and consists of multiple thermocouples connected in series. There are 32 pairs of thermocouples placed around the center of membrane and each thermocouple is designed with two kinds of material, n^+^Poly and metal. The material of Metal 1–Metal 4 layers is aluminum, and titanium is used as contact layers to connect these metal layers. Four quarters of sensing units of thermopile were connected and the sensing area at the center will give a temperature difference. According to the Seebeck effect, serial connected thermocouples with two different materials will produce a weak voltage difference between the hot and cold junctions after being irradiated with thermal radiation. The Seebeck coefficient of the thermoelectric element combined with two different materials (Metal1, n^+^Poly) is 119 μV/K and the resistivity of the n^+^Poly layer is 0.85 mΩ-cm. The two output electrodes for each sensing unit can be independently connected to the amplifier and they can be also serial connected by four breakers of specific design. 

**Figure 4:**
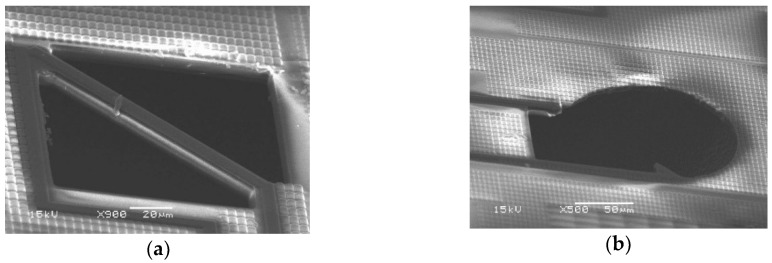
(**a**) SEM of a circuit breaker; (**b**) SEM of a circuit breaker with damaged bridge.

**Figure 5:**
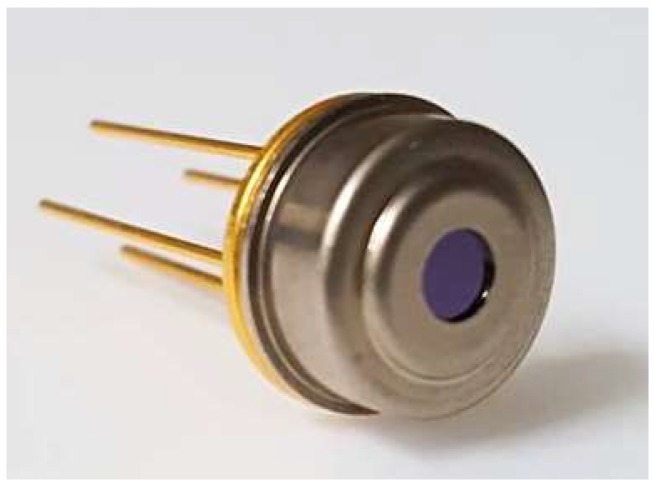
Photography of the TO-5 metal can package which the thermopile is sealed and die-bonding inside.

In this chip, the circuit breaker is designed to select which units to be serial connected as shown in Figure 4. The breaker is designed with a floating bridge which its underneath of silicon is removed away. There is a metal interconnection running over the bridge which can be opened by manual damage. The total resistance of the structure with four units is measured to be about 14.8 kΩ and the resistance of each unit is about 3.7 kΩ. In order to improve the stability and accuracy of the sensor for thermal radiation measurement, the chip is sealed with a TO-5 metal can package, as shown in Figure 5.

## 4. Operation of Dual Mode Modulation

Beyond conventional sensing architecture, we propose a new infrared temperature sensing approach based on a single thermopile sensor with dual modes switching. Usually, the thermopile works as a thermoelectric sensor and the material properties of thermoelectric sensor are also sensitive to temperature, which means that it can also be used as a thermistor for ambient compensation. Nevertheless, for most of the applications, the temperature rise induced by the infrared radiation on the membrane of thermopile sensor is expected less than 0.1 °C which means the temperature of thermopile elements is also adequate and accurate to monitor the ambient temperature. Therefore, the thermopile can be considered to be both a thermoelectric sensor and thermistor.

### 4.1. Thermopile Sensing Mode

**Figure 6:**
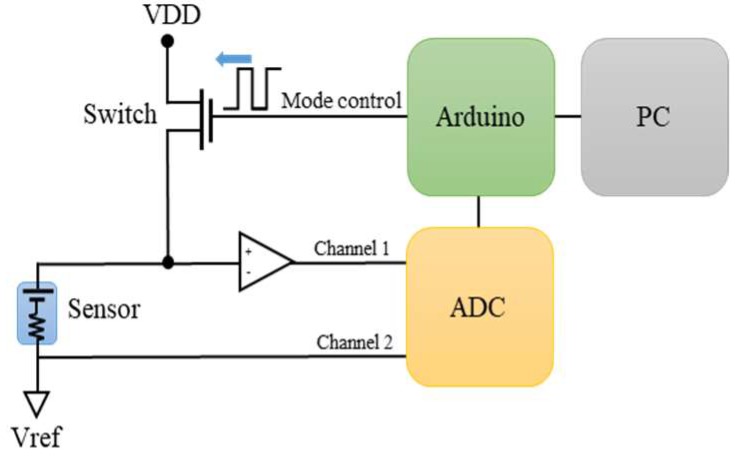
Architecture of proposed dual modes circuit for thermopile sensor.

For the circuit architecture, in order to achieve dual mode switching in a single circuit, a PMOS is placed at the positive input of the OP whereas the PMOS is turned on or off through the Arduino. As shown in Figure 6, when the PMOS is off, it means the switch is open and the signal from the thermopile sensor is delivered to the positive input of amplifier AD8551 (Norwood, MA, USA). Therefore, the sensing circuit performs the amplification and measurement of thermopile. Nevertheless, it will take a certain time for the signals between the switching to reach a steady state. The time for switching is the major factor for the sampling time of system, which will be analyzed thoroughly on the conditions of resistor–capacitor (RC) time constant. 

### 4.2. Effective Thermistor Sensing Mode

**Figure 7:**
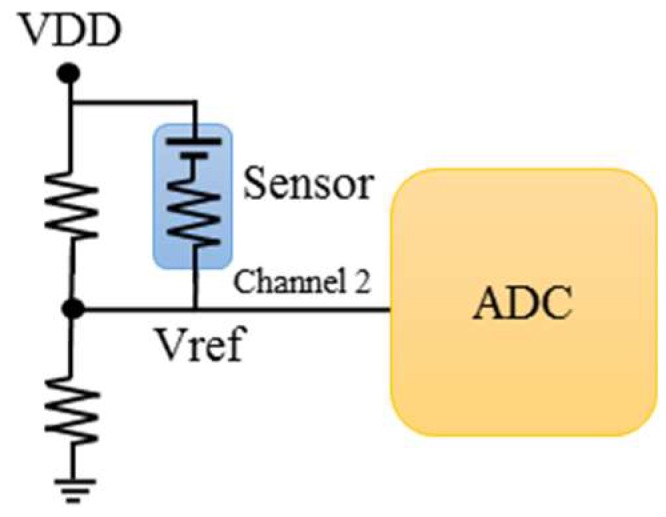
Operation of effective thermistor sensing mode with PMOS on-state.

For the mode, the sensor is simply considered as a resistor which acts as an effective thermistor which responses to the ambient temperature. When the PMOS is turned on, the circuit is switched to the effective thermistor sensing mode and an electrode of the thermopile sensor is pulled to Vdd. Since the sensor is connected in parallel with other resistors, and the divided voltage of the series circuit is used as the measurement signal Vref of the effective thermistor. As shown in Figure 7, finally, another channel of the ADC is used to read the voltage signal Vref and it is proved that the circuit can be switched to achieve the purpose of different measurements. This data acquisition system is designed with an embedded Atmel SAM3X8E ARM Cortex-M3 micro-controller (Chandler, AZ, USA), to control the circuit and communication with PC.

## 5. Results and Analysis

In order to investigate the stability of the circuit, the sampling time of the signal needs to be investigated, including the response time of the sensor and the electronic component, and the time constant of the filter. These issues will be analyzed and studied separately in the following.

### 5.1. Frequency Response and Thermal Time Constant for Sensor

Based on the thermal circuit analysis, the thermal behavior is formulated with the heat equation, Equation (6) and the thermal time constant is derived. There are several factors and methods to express the speed of response for sensor. The cut-off frequency, *f_c_* of the frequency response curve through the modulation of infrared radiation with mechanical chopper is first proceeded and then it can conduct the thermal time constant expressed as the following Equation (7). H is the heat capacitance of thermopile membrane and *G* is the thermal conductance including the solid and convection conduction to the environment with ambient temperature *T_a_*.

(6) $$H\frac{dT}{dt}+G(T-T_{a})=\Delta \Phi ,$$

(7) $$\tau =\frac{H}{G}=\frac{1}{\omega_{c}}=\frac{1}{2\pi f_{c}},$$

The results of the infrared modulation measurement for thermal time constants of the sensor under various conditions are shown in Table 1.

### 5.2. Investigation on Transient Response and Sensing Characteristics for Circuit

#### 5.2.1. Relationship between Transition Time and Jitter

**Figure 8:**
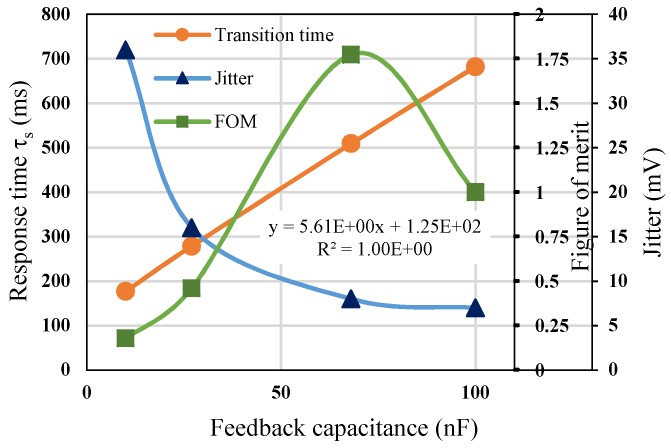
Figure of merit for dual modes operation.

Since the response time of the circuit is affected by the RC filtering, the resistance *R* is related to the gain of amplification. Therefore, we analyze the response time for different feedback capacitors (10 nF, 27 nF, 68 nF, 100 nF). The simulation results show that the faster the response time, the greater the amount of noise, as shown in Figure 8. The opposite relationship between the response time and the amount of noise makes it difficult to determine the proper capacitance in the circuit. Therefore, the figure of merit (FOM) is expressed as the ratio of speed of sampling rate to noise (jitter) to explore the optimal conditions for RC. 

According to the simulation results, the optimal condition of feedback capacitance is 68 nF which gives the maximum FOM of 1.75. It means that the output voltage will have a lower noise jitter with 8 μV and an acceptable response time with 440 ms for infrared temperature measurement, for example, tympanic thermometers and industrial infrared thermometers. 

#### 5.2.2. Simulation vs. Measurement

**Figure 9:**
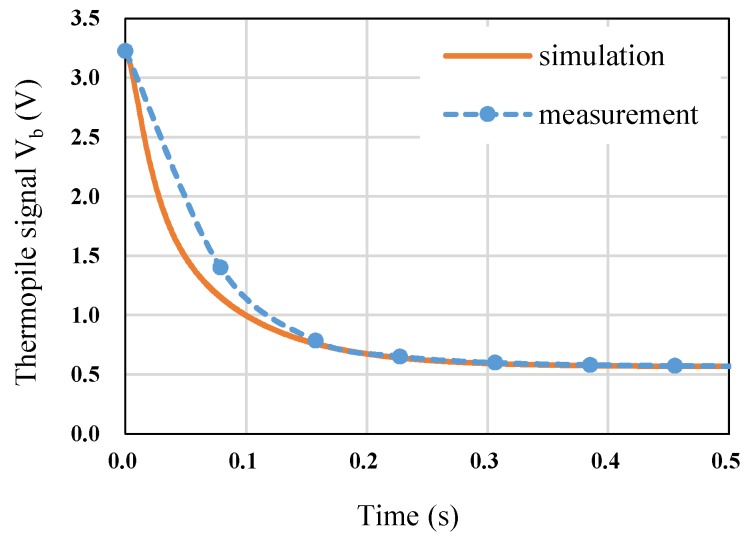
Simulation vs. measurement for switching from effective thermistor sensing mode to thermopile sensing mode.

After finding the best condition of the feedback capacitor (68 nF), we compared the transient response in the simulation and measurement. The result is shown in Figure 9. The trends of the transient time are in good agreement for both the measurement and the simulation.

#### 5.2.3. Analysis of Transition Performance of System

To analyze the transition performance of system, the transition time of the signal is needed to be investigated between the dual mode of modulation. These factors will be estimated and discussed separately in the following. The response time of the overall performance of system includes the time constants for a sensor, an amplifier, and a switching circuit, corresponding to $\tau_{s}$, $\tau_{amp}$, and $\tau_{sw}$, respectively, and is formulated as follows:

(8) $$\tau =\tau_{s}+\tau_{amp}+\tau_{sw}\approx \tau_{sw},$$

The thermal time constant of the sensor is about 4.8 ms, the slew rate of the amplifier AD8551 is 0.4 V/ms, and the transition time of the switching circuit is about 440 ms. The time required to switch the circuit is much longer than the other component characteristics, so the response time is simplified as Equation (8).

### 5.3. Measuerment Result for Dual Mode Switching Circuit

#### 5.3.1. Calibration of Thermal Radiation for Dual Mode Operation

**Figure 10:**
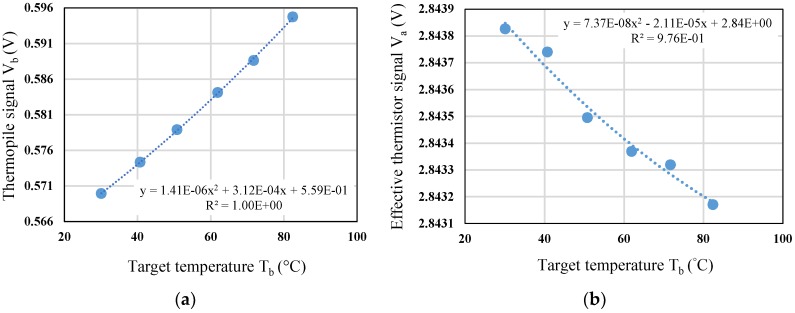
(**a**) Calibration of thermopile vs. target temperature; (**b**) Effective thermistor voltage vs. target temperature during calibration.

In thermal radiation measurement, the target temperature is set from 30 to 80 °C. In Figure 10a, the sensor output voltage rises as the target temperature increases, and its sensitivity is about 0.5 mV/°C for target temperature change. After switching to the effective thermistor sensing mode, the voltage of effective thermistor is acquired and analyzed, and the drop of voltage range is about 0.6 mV during the calibration, as shown in Figure 10b.

#### 5.3.2. Calibration of Effective Thermistor Sensing

**Figure 11:**
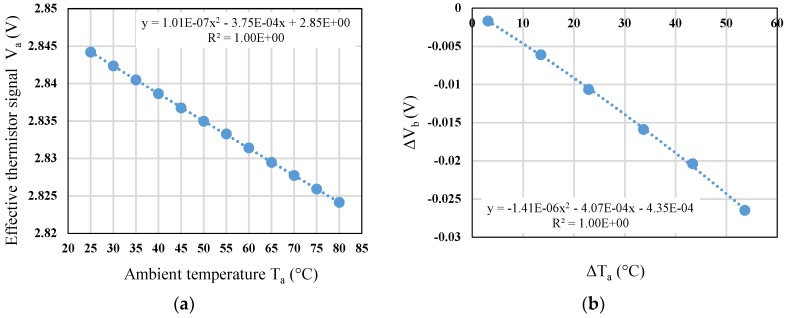
(**a**) Calibration of effective thermistor vs. ambient temperature; (**b**) The change of thermopile output voltage vs. the change of ambient temperature.

In general, when performing thermal radiation measurement, the sensor is affected by the variation of ambient temperature in addition to the target temperature. It means that the radiation of blackbody will cause a drop of output voltage of thermopile while the sensor is heated during the measurement. Therefore, temperature calibration and ambient temperature compensation are necessary for the measurement of sensor. Figure 11a show the calibration curve of the effective thermistor, and the ambient temperature range is setup from 25 to 80 °C with a step of 5 °C. From Figure 11a, the sensitivity is derived as −0.4 mV/°C for ambient temperature change. Then, the relationship between the change of ambient temperature Δ*T_a_* and the change of thermopile output voltage Δ*V_b_* is measured and analyzed, as shown in Figure 11b.

#### 5.3.3. Investigation of Thermopile Sensing with Ambient Temperature Compensation

**Figure 12:**
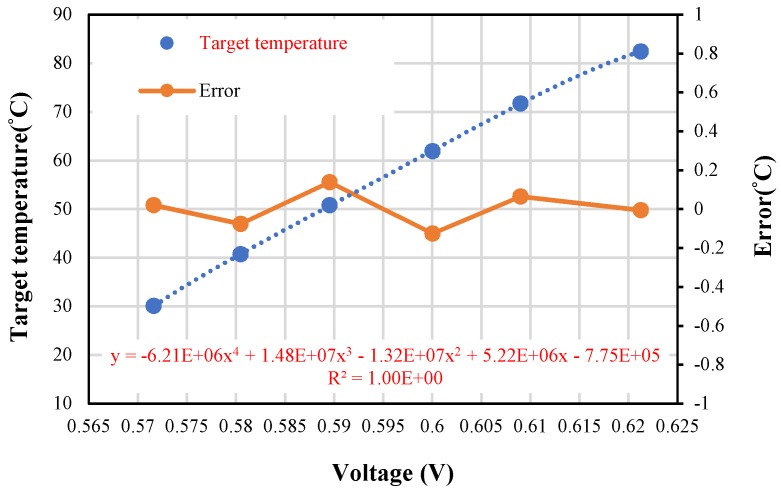
Calibration of thermopile vs. target temperature with ambient temperature.

During the thermopile calibration, the effective thermistor is used to compensate the ambient temperature difference to guarantee thermal radiation exchange between the standard blackbody and thermopile sensor. Then we adjust the standard blackbody with temperature from about 30 °C to 80 °C and measure the thermopile output voltage under a stable environment. The calibration is proceeded under dual mode switching, measurement of the target temperature is achieved and a calibration curve after the ambient temperature compensation is shown in Figure 12. The curve of data is fitted with 4th order of polynomial, which is in agreement of Stefan–Boltzmann’s law. The analysis of errors generated during the measurement and calculation comes from the instability of ambient temperature compensation. The maximum error of ambient temperature compensation from curve fitting in Figure 11b is calculated about 0.158 mV for the worst case Δ*T_a_* = 53 °C, and it will deduce the maximum error of target temperature with 0.17 °C. Finally, for the measurement of target temperature from 30 °C to 80 °C and the ambient temperature drift Δ*T_a_* = 1.8 °C, the overall error is within ±0.14 °C. It is reasonable and practical for applications based on the dual mode modulation technique which meets the American society for testing materials (ASTM) standard.

## 6. Conclusions

For precision measurement of remote temperature, the major key of infrared technology is built on the calibration of target temperature and compensation of ambient temperature with two different kinds of sensor. Superior to the traditional sensing architecture with two sensors of infrared sensor and thermistor, we propose a novel sensing approach based on a single thermopile sensor with dual modes modulation which play the roles of both infrared sensing and ambient temperature sensor. A switching and sensing circuit is proposed and realized with a chopper amplifier AD8551 and PMOS for switching between detection of thermal radiation from the target and the ambient temperature for compensation. The signal of infrared sensor after amplification and low-pass filtering is sent to the one of the analogy input channel of ADC AD7799. Under switching of PMOS, the voltage signal of resistance of thermopile sensor is picked up into the second channel of the ADC. Both signals from switching circuit are sent to the PC via the serial peripheral interface (SPI) interface of the ADC. After temperature calibration of thermopile resistance, the temperature coefficient of resistance (TCR) of our proposed sensor is about 0.093%/°C. The thermal time constant of proposed thermopile is calibrated at around 4.80 ms. The calibration is proceeded under dual mode switching, measurement of the target temperature is achieved, and a calibration curve is derived with the ambient temperature compensation. 

After carefully calibrating and validating several experimental conditions, our proposed infrared temperature measuring with dual modes modulation shows excellent temperatures in agreement with the measured data. Further, the target temperature can be compensated by the effective thermistor, and the estimated error is estimated less than 0.14 °C.

## Figures and Tables

**Table 1:** Frequency response measurement with infrared radiation modulation.

Target Temperature (K)	Relative Cut-off Frequency (Hz)	Relative Thermal Time Constant (ms)
373.15	1	1
473.15	85.5	1.17
573.15	83.7	1.20
673.15	78.0	1.29

The reference cut-off frequency is 38.7 Hz. The reference thermal time constant is 4.1 ms. Average of thermal time constant *τ*: 4.8 ms.
